# Crystal structure of 4-bromo-*N*-(propyl­carbamo­yl)benzene­sulfonamide

**DOI:** 10.1107/S2056989022003723

**Published:** 2022-04-07

**Authors:** Mustafa Bookwala, Saloni Patel, Patrick T. Flaherty, Peter L. D. Wildfong

**Affiliations:** a Graduate School of Pharmaceutical Sciences, Duquesne University, 600 Forbes Avenue, Pittsburgh, PA 15282, USA

**Keywords:** bromo­propamide, crystal structure, single-crystal XRD, structural analogue

## Abstract

The title compound, C_10_H_13_BrN_2_O_3_S, **1**, contains a sulfonyl urea moiety, which possesses potential therapeutic functions (*e.g.*, anti-diabetic and herbicidal). The geometry of **1** is similar to its closely related analogues, chlorpropamide and tolbutamide. This compound crystallizes in the monoclinic space group *C*2/*c*, having one mol­ecule in its asymmetric unit. The crystal structure of **1**, recorded at 296 K, shows inter­molecular N—H⋯O and C—H⋯O-type infinite hydrogen-bonded chains involving the sulfonyl urea moiety. Hirshfeld surface analysis and the two-dimensional fingerprint plots confirmed hydrogen bonding as the dominant feature in the crystal packing.

## Abstract 2

The X-ray crystal structure, and Hirshfeld surface analysis of a sulfonyl urea analogue, 4-bromo-*N*-(propyl­carbamo­yl)benzene­sulfonamide is reported.

## 1. Chemical context

The title compound, **1**, also known as bromo­propamide, is a sulfonyl urea structural analogue, whose chemical structure is shown in the scheme. Compounds containing sulfonyl urea as the structural core have been used extensively for the treatment of Type II diabetes (McLamore *et al.*, 1959 ▸), by stimulating insulin secretion from pancreatic β-cells by binding to the ATP-sensitive potassium channel (Proks *et al.*, 2002 ▸). Additionally, sulfonyl urea structural analogues have shown therapeutic action as herbicides and diuretic agents (Tanwar *et al.*, 2017 ▸). Thus, the title compound was synthesized in order to perform biological characterization. The crystal structures of several sulfonyl urea compounds have been reported, especially mol­ecules closely related to **1** that contain the *N*-carbamoyl­benzene­sulfonamide substructure, all of which have multiple polymorphic forms (Kimura *et al.*, 1999 ▸; Drebushchak *et al.*, 2006 ▸; Fedorov *et al.*, 2017 ▸). Subtle changes to the mol­ecule have shown drastic effects on its biological activity and also the arrangement of mol­ecules in the crystal structure (Bieszczad *et al.*, 2020 ▸). Thus, it is of inter­est to not only confirm the mol­ecular structure of bromo­propamide, but to also identify its crystal packing relative to other structural analogues. 
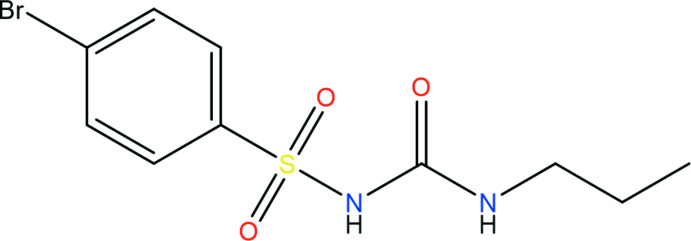


## 2. Structural commentary

**Figure 1:**
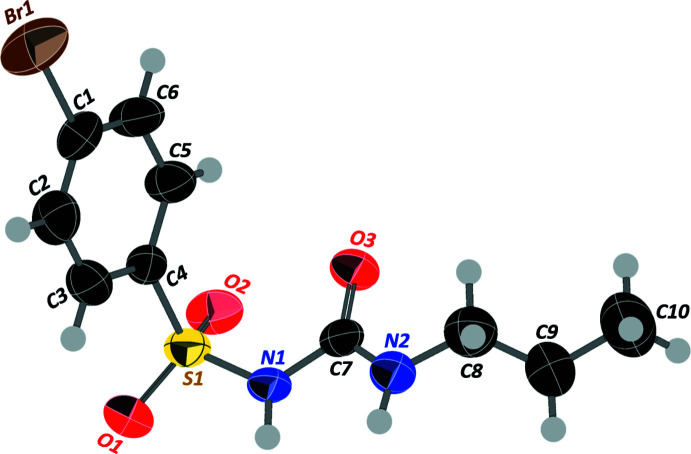
The mol­ecular structure of 4-bromo-*N*-(propyl­carbamo­yl)benzene­sulfonamide with atomic numbering scheme. Displacement ellipsoids are drawn at the 50% probability level.

Bromo­propamide crystallizes in the centrosymmetric and achiral monoclinic space group *C*2/*c*, having one mol­ecule in the asymmetric unit (Fig. 1 ▸). The Br1—C1 bond length [1.887 (2) Å] is in good agreement with other structures containing a bromo­phenyl moiety (Khamees *et al.*, 2019 ▸; Arif Tawfeeq *et al.*, 2019 ▸). The bond length between C1—C2 [1.363 (4) Å], is the shortest among all the bond lengths in the phenyl group, possibly due to the inductive effect of bromine. The brominated phenyl ring is almost perpendicular [C4—S1—N1 = 105.65 (11)°] to the sulfonyl urea *n*-propyl group, resulting in an L-shaped mol­ecular structure. This is similar to chlorpropamide, a structural analogue of **1** [Cambridge Structural Database (CSD; Groom *et al.*, 2016 ▸) refcode: BEDMIG10; 105.87°; Drebushchak *et al.*, 2009 ▸). The sum of the bond angles around N1 and N2 is 360°, indicating *sp^2^* hybridization, caused by the delocalization of the lone electron pair of N1 and N2 into the π bond of the carbonyl group. This is also supported by the trigonal-planar mol­ecular geometry of C7—N1—S1 [123.94 (17)°], C7—N1—H1 (118°), S1—N1—H1 (118°), C7—N2—C8 [123.5 (2)°], C7—N2—H2 (118.3°), and C8—N2—H2 (118.3°). The C7—N2 bond length is 1.319 (3) Å, which is lower than the typical range; however, the values are similar to those in the crystal structures of bromo­propamide analogues, chlorpropamide (1.315 Å; CSD refcode: BEDMIG14; Drebushchak *et al.*, 2009 ▸) and tolbutamide (1.319 Å; CSD refcode: ZZZPUS13; Drebushchak *et al.*, 2011 ▸). The propyl chain takes the stable *trans* conformation so as to have a maximum distance of 3.794 Å between N2 and C10, while C10 exhibits rotational disorder, possibly due to the X-ray diffraction experiments being conducted at 296 K. Overall, the crystal structure of **1** showcases bond lengths (Allen *et al.*, 1987 ▸) and angles typical of the expected ranges.

## 3. Supra­molecular features

**Figure 2:**
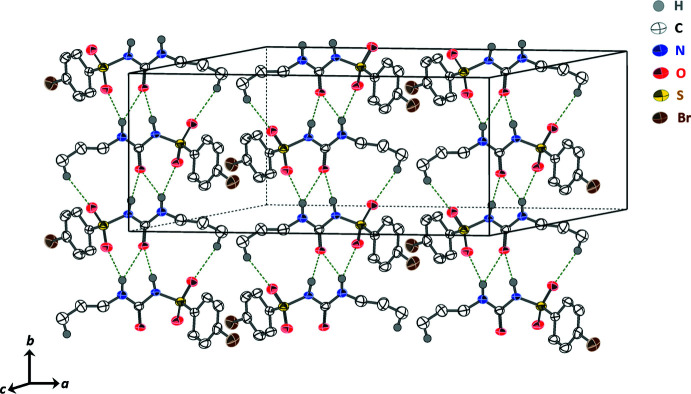
Infinite hydrogen-bonding involving the sulfonyl urea moiety in 4-bromo-*N*-(propyl­carbamo­yl)benzene­sulfonamide. Hydrogen bonding between N1—H1⋯O2, N2—H2⋯O2, N2—H2⋯O3, and C10—H10*C*⋯O1 is shown as green dotted lines. Displacement ellipsoids are drawn at the 30% probability level. Only H atoms involved in hydrogen bonding are shown.

**Figure 3:**
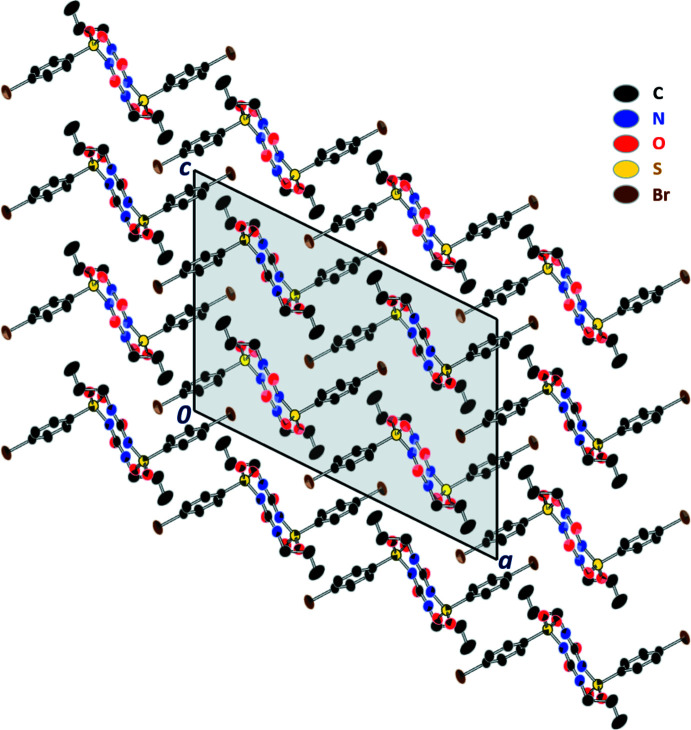
The crystal packing in 4-bromo-*N*-propyl­carbamo­yl)benzene­sulfonamide viewed along the *b* axis. Anti­parallel stacking of the bromo­phenyl has a centroid-to-centroid distance of 4.213 Å. Displacement ellipsoids are drawn at the 30% probability level. H atoms are not shown for clarity.

The crystal packing of the title compound is dominated by hydrogen bonding, which is shown in Fig. 2 ▸. Geometric details of the hydrogen bonds are listed in Table 1 ▸. Inter­molecular N—H⋯O-type hydrogen bonds link the mol­ecules into infinite chains, which stretch along the *b*-axis direction (Fig. 2 ▸). Hydrogen bonding between the H1 and O3 atoms of neighboring mol­ecules have distances of H1⋯O3 = 1.94 Å, N1⋯O3 = 2.791 (3) Å. The strongest of these is N1—H1⋯O3, with an angle of 171.9°, followed in rank-order of strength by the hydrogen-bonds between H2⋯O2 = 2.24 Å, N2⋯O2 = 2.998 (3) Å (angle of 146.8° between N2—H2⋯O2) and H2⋯O3 = 2.642 Å, N2⋯O3 = 3.351 (3) Å (angle of 140.6° between N2—H2⋯O3). Additionally, weak C—H⋯O type hydrogen bonds also help, to some extent, with the mol­ecular packing. The inter­molecular distance between H10*C*⋯O1 is 2.61 Å; C10⋯O1 is 4.028 Å with an angle of 173.7° between C10—H10*C*⋯O1. The distances and angles of the C—H⋯O-type hydrogen bond observed in the present structure are within the reported ranges (Desiraju, 1991 ▸; Gumireddy *et al.*, 2021 ▸). Overall, the atoms involved in hydrogen bonding for bromo­propamide are identical to those in the crystal structure for its analogue chlorpropamide (CSD refcode: BEDMIG10; Drebushchak *et al.*, 2009 ▸). Fig. 3 ▸ shows the unit cell of the title compound along the *b*-axis. It appears that the anti-parallel flanked phenyl rings are stacked. However, a centroid-to-centroid distance of 4.213 (2) Å, which is outside the range of π–π stacking inter­actions (Chulvi *et al.*, 2015 ▸; Ahmed *et al.*, 2019 ▸), supports its absence.

## 4. Hirshfeld surface analysis

**Figure 4:**
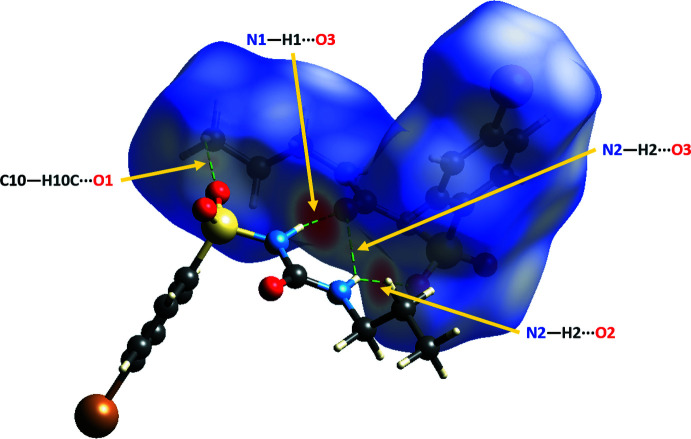
Hirshfeld surface of 4-bromo-*N*-(propyl­carbamo­yl)benzene­sulfonamide mapped over *d*_norm_, displays close contacts in the crystal. The non-covalent inter­actions indicated by the red spots are labeled.

**Figure 5:**
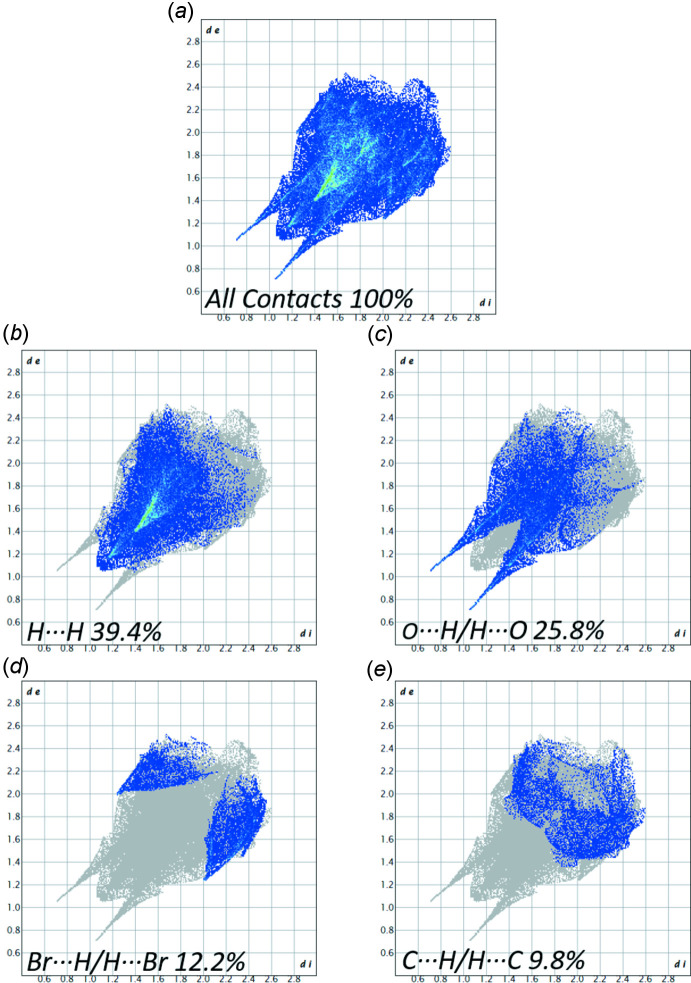
The two-dimensional fingerprint plots of 4-bromo-*N*-(propyl­carbamo­yl)benzene­sulfonamide with their relative contribution to the Hirshfeld surface. The units of *d*_i_ and *d*_e_ are Å.

Hirshfeld surface analysis was carried out using *CrystalExplorer17.5* (Turner *et al.*, 2017 ▸; Spackman *et al.*, 2021 ▸) mapped over *d*_norm_, which was estimated by the calculations of the external and inter­nal distances to the nearest nucleus and built over a volume of 322.24 Å^3^ having an area of 304.35 Å^2^, with scaled color of −0.6347 a.u. (red) to 1.2043 a.u. (blue). The Hirshfeld surface of **1**, shown in Fig. 4 ▸, displays close contacts between N1—H1⋯O3, N2—H2⋯O2, N2—H2⋯O3, and C10—H10*C*⋯O1, supporting the conclusions about hydrogen-bonding inter­actions. Hirshfeld surfaces and their associated two-dimensional fingerprint plots were used to qu­antify the various inter­molecular inter­actions. The overall two-dimensional fingerprint plot for bromo­propamide (Fig. 5 ▸ *a*) and those delineated into major contacts: H⋯H, O⋯H/H⋯O, Br⋯H/H⋯Br, and C⋯H/H⋯C are shown in Fig. 5 ▸ *b*–*e*. The other contacts have lower contributions, with individual contributions <4.3% and a sum <12.8%. The H⋯H inter­atomic contacts, which appear as a single spike in the center at *d*_e_ = *d*_i_ = 1.1 Å (Fig. 5 ▸ *b*), generated 39.4% of the Hirshfeld surface, denoting these contacts have a significant effect on the mol­ecular packing. The O⋯H/H⋯O inter­atomic contacts, which appear as a pair of spikes with tips at *d*_e_ + *d*_i_ ∼1.75 Å (Fig. 5 ▸ *c*), represent 25.8% of the total surface and confirms the prominent role of multiple hydrogen bonds in the mol­ecular arrangement within the crystal structure. Br⋯H/H⋯Br and C⋯H/H⋯C contribute 12.2% and 9.8%, respectively, to the Hirshfeld surface. The placement of mol­ecules in the crystal structure of the title compound results in efficient packing, as seen in the Hirshfeld surface analysis, which is further supported by the crystallographic density of 1.626 g cm^−3^, which is relatively higher than other small mol­ecule organic compounds (Bookwala *et al.*, 2020 ▸, 2022 ▸).

## 5. Database survey

A search in the Cambridge Structural Database (Version 5.41, update of March 2020; Groom *et al.*, 2016 ▸) for compounds possessing the sulfonyl urea substructure resulted in 178 hits, reinforcing the importance of this scaffold as having potential as an anti-diabetic or diuretic drug, and a herbicide. Of the 178 hits, 82 were distributed among chlorpropamide (deposited structures: 20), tolaza­mide (deposited structures: 40), and tolbutamide (deposited structures: 22), all of which share a close structural relationship to bromo­propamide. The search was then narrowed to identify compounds containing *N*-(propyl­carbamo­yl)benzene­sulfonamide, which resulted in identification of only chlorpropamide polymorphs, confirming the absence of reported crystal structures for analogues having different halogen substitutions. An exact search for the title compound resulted in zero hits, further supporting the previous claim. Thus, X–ray studies were important to identify, if any, changes in the crystal structure by replacing the peripheral Cl with a Br atom.

## 6. Synthesis and crystallization

**Figure 6:**
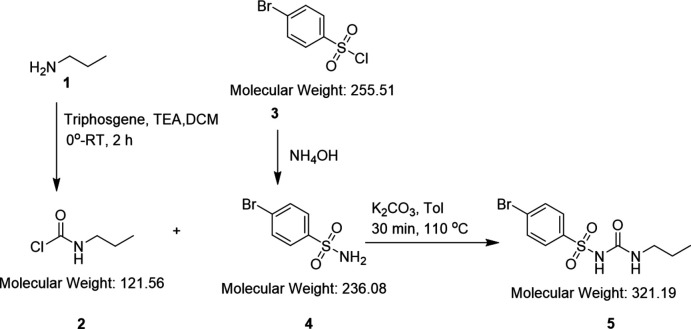
Reaction scheme for the synthesis of 4-bromo-*N*-(propyl­carbamo­yl)benzene­sulfonamide.

The synthesis of 4-bromo-*N*-(*n*-propyl­carbamo­yl)benzene­sulfonamide used *in situ* formation of *n*-propyl­iso­cyanate from *n*-propyl­carbamic chloride with direct capture by 4-bromo­benzene­sulfonamide in the presence of excess potassium carbonate in refluxing toluene (Fig. 6 ▸). This is a new methodology to generate sulfonyl ureas in an atom-efficient manner with identical chemical characterization to prior methods proceeding *via* carbamate (Marshall & Sigal, 1958 ▸) or carbon­ate (Tanwar *et al.*, 2017 ▸) inter­mediates. A manuscript describing the optimization of this synthetic strategy is in preparation.

**n-Propyl­carbamic chloride** (labeled **2** in Fig. 6 ▸): A solution of triphosgene (2.24 g, 22.62 mMol as phosgene) in 25 mL of di­chloro­methane (DCM) was cooled in a 100 mL round-bottom flask. A solution of tri­ethyl­amine (TEA) (5.6 mL, 40 mMol), *n*-propyl­amine (labeled **1** in Fig. 6 ▸) (1.7 mL, 20.1 mMol) and 10 mL of DCM was added to the triphosgene solution with slow dropwise addition over 15 min maintaining an inter­nal temperature between 278 and 283 K. The cooling bath was removed following addition and the reaction was permitted to stir for an additional 2 h at 296 K. The reaction mixture was cooled in an ice/water bath and then transferred to a 125 mL separatory funnel previously cooled in ice–water. The mixture was then washed with 3 × 5 mL portions of ice-cold water, 2 × 5 mL of ice-cold 0.5 *N* HCl, 2 × 5 mL portions of ice-cold brine, dried Na_2_SO_4_, deca­nted, and the solvent was carefully removed under reduced pressure without heating to theoretical mass. The conversion to *n*-propyl­carbamic chloride was confirmed with IR absorbance of 1734 cm^−1^ and afforded 2.5 g (98% of a light yellow oil) and stored at 253 K until use.

**4-Bromo­benzene­sulfonamide** (labeled **4** in Fig. 6 ▸): Synthesized using a variation of the published procedure (Anana *et al.*, 2006 ▸). Concentrated NH_4_OH (150 mL, 1.10 mol) was charged into a 500 mL three-neck round-bottom flask equipped with an overhead stirrer, thermowell, and condenser. The reaction was then cooled in an ice/water bath to an inter­nal temperature of 283 K. Solid 4-bromo­benzene­sulfonyl chloride (49.9954 g, 0.1957 mol) was added in portions over 5 min. The ice/water bath was removed and the mixture was stirred at room temperature for 15 min and then brought to 308 K for 30 min. After this, the reaction was warmed to reflux for an additional 30 min. The reaction was followed by thin-layer chromatography (TLC) [*R*_f_ = 0.69 (labeled **3** in Fig. 6 ▸), *R*_f_ = 0.54 (labeled **4** in Fig. 6 ▸) 1/1 hexa­ne/ethyl acetate (H/EA), short wavelength ultra-violet (SWUV)]. The reaction was cooled to room temperature upon consumption of the starting material and then poured into 200 mL of ice-cold water. This heterogeneous mixture was brought to pH = 1 (pHydrion paper) with 6 *N* HCl. The precipitated white solid was collected on a #1 Whatman filter paper, pressed dry with a rubber dam, and dried 12 h in a drying pistol (P_2_O_5_, 150 mTorr, 383 K) to afford 43.03 g (93.5%) of a white solid. Proton identical with literature (Richardson *et al.*, 2007 ▸), m.p. 434–438 K (m.p. lit: 435 K).

**4-Bromo-** ***N*** **-(** ***n*** **-propyl­carbamo­yl)benzene­sulfonamide** (labeled **5** in Fig. 6 ▸): *n*-Propyl­carbamic chloride (labeled **2** in Fig. 6 ▸), (2.0 g, 15.9 mMol), toluene (15 mL), K_2_CO_3_, (2.019 g, 14.6 mMol), and 4-bromo­benzene­sulfonamide (labeled **4** in Fig. 6 ▸), (1.4306 g, 6.06 mMol) were added to a dry 100 mL round-bottom flask fitted with a straight condenser and brought to reflux for 30 min. Upon loss of the sulfonamide (TLC: *R*_f_ = 0.86, 1/1: H/EA SiO_2_, SWUV, I_2_), the heating was stopped, the oil bath was removed, and the reaction was permitted to cool to room temperature. The resulting white suspension was cooled in an ice/water bath and brought to a pH = 1 (pHydrion paper: red) with 6 *N* HCl. This mixture was extracted with 3 × 10 mL portions of EA, washed [3 × 5 mL 1 *N* HCl, then 2 × 5 mL NaCl (sat, aq.)], dried Mg_2_SO_4_, filtered under vacuum through #1 Whatman filter paper, and then the solvent was removed under reduced pressure to give 2.2 g of a white solid. This material was purified on a SiO_2_ column (1/1: H/EA SiO_2_, *R*_f_ = 0.66) then recrystallized from toluene to yield, after drying in a drying pistol at 383 K (P_2_O_5_, 150 µTorr), 0.87 g (41%) of fine white crystals. ^1^H NMR was identical to prior synthesis (Tanwar *et al.*, 2017 ▸), m.p. 411 K (m.p. lit: 406–408 K).

Crystals obtained from toluene were very small; therefore, they were dissolved in methanol to obtain a supersaturated solution (37.5 mg mL^−1^). This was placed in a 20 mL scintillation vial, which was covered with Parafilm^®^ and punched with 5 pin holes to allow slow evaporation of methanol at room temperature over several days, until larger single crystals appeared.

## 7. Refinement

Crystal data, data collection and structure refinement details are summarized in Table 2 ▸. H atoms were positioned geometrically (aromatic C—H = 0.93 Å, amide N—H = 0.86 Å methyl­ene C—H = 0.98 Å, and methyl C—H = 0.96 Å) and treated as riding atoms during refinement, with *U*_iso_ (H) = 1.2*U*_eq_(aromatic C, amide N, and methyl­ene C) or 1.5*U*_eq_(methyl C). The methyl groups were allowed to rotate about their local threefold axes.

## Supplementary Material

Crystal structure: contains datablock(s) I. DOI: 10.1107/S2056989022003723/mw2187sup1.cif

Structure factors: contains datablock(s) I. DOI: 10.1107/S2056989022003723/mw2187Isup2.hkl

Supporting information file. DOI: 10.1107/S2056989022003723/mw2187Isup3.cml

CCDC reference: 2164397

Additional supporting information:  crystallographic information; 3D view; checkCIF report

## Figures and Tables

**Table 1:** Hydrogen-bond geometry (Å, °)

*D*—H⋯*A*	*D*—H	H⋯*A*	*D*⋯*A*	*D*—H⋯*A*
N1—H1⋯O3^i^	0.86	1.94	2.791 (3)	172
N2—H2⋯O2^i^	0.86	2.24	2.998 (3)	147
N2—H2⋯O3^i^	0.86	2.64	3.351 (3)	141

Symmetry code: (i) 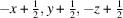 .

**Table 2:** Experimental details

Crystal data	
Chemical formula	C_10_H_13_BrN_2_O_3_S
*M*_r_	321.19
Crystal system, space group	Monoclinic, *C*2/*c*
Temperature (K)	296
*a*, *b*, *c* (Å)	21.0939 (12), 9.2520 (6), 15.0283 (10)
β (°)	116.211 (4)
*V* (Å^3^)	2631.4 (3)
*Z*	8
Radiation type	Mo *K*α
μ (mm^−1^)	3.28
Crystal size (mm)	0.25 × 0.12 × 0.05

Data collection	
Diffractometer	Bruker SMART APEXII
Absorption correction	Multi-scan (*SADABS*; Krause *et al.*, 2015 ▸)
*T*_min_, *T*_max_	0.522, 0.746
No. of measured, independent and observed [*I* > 2σ(*I*)] reflections	15567, 2918, 1776
*R*_int_	0.047
(sin θ/λ)_max_ (Å^−1^)	0.643

Refinement	
*R*[*F* ^2^ > 2σ(*F* ^2^)], *wR*(*F* ^2^), *S*	0.036, 0.095, 1.01
No. of reflections	2918
No. of parameters	155
H-atom treatment	H-atom parameters constrained
Δρ_max_, Δρ_min_ (e Å^−3^)	0.26, −0.26

Computer programs: *SMART* and *SAINT* (Bruker, 1998 ▸), *SHELXS97* (Sheldrick, 2008 ▸), *SHELXL2018/3* (Sheldrick, 2015 ▸), and *CrystalMaker* (Palmer, 2014 ▸).

## supplementary crystallographic information

### Crystal data

**Table:**

C_10_H_13_BrN_2_O_3_S	*D*_x_ = 1.622 Mg m^−^^3^
*M**_r_* = 321.19	Melting point: 411 K
Monoclinic, *C*2/*c*	Mo *K*α radiation, λ = 0.71073 Å
*a* = 21.0939 (12) Å	Cell parameters from 2918 reflections
*b* = 9.2520 (6) Å	θ = 2.2–27.2°
*c* = 15.0283 (10) Å	µ = 3.28 mm^−^^1^
β = 116.211 (4)°	*T* = 296 K
*V* = 2631.4 (3) Å^3^	Rectangular Plate, colorless
*Z* = 8	0.25 × 0.12 × 0.05 mm
*F*(000) = 1296	

### Data collection

**Table:**

Bruker SMART APEXII diffractometer	1776 reflections with *I* > 2σ(*I*)
Radiation source: Fine-focus Sealed Tube	*R*_int_ = 0.047
φ and ω Scans scans	θ_max_ = 27.2°, θ_min_ = 2.2°
Absorption correction: multi-scan (SADABS; Krause *et al.*, 2015)	*h* = −26→27
*T*_min_ = 0.522, *T*_max_ = 0.746	*k* = −11→11
15567 measured reflections	*l* = −19→19
2918 independent reflections	

### Refinement

**Table:**

Refinement on *F*^2^	Primary atom site location: structure-invariant direct methods
Least-squares matrix: full	Secondary atom site location: difference Fourier map
*R*[*F*^2^ > 2σ(*F*^2^)] = 0.036	Hydrogen site location: inferred from neighbouring sites
*wR*(*F*^2^) = 0.095	H-atom parameters constrained
*S* = 1.01	*w* = 1/[σ^2^(*F*_o_^2^) + (0.0397*P*)^2^ + 0.7765*P*] where *P* = (*F*_o_^2^ + 2*F*_c_^2^)/3
2918 reflections	(Δ/σ)_max_ = 0.001
155 parameters	Δρ_max_ = 0.26 e Å^−^^3^
0 restraints	Δρ_min_ = −0.26 e Å^−^^3^

### Special details

**Table:**

Geometry. All esds (except the esd in the dihedral angle between two l.s. planes) are estimated using the full covariance matrix. The cell esds are taken into account individually in the estimation of esds in distances, angles and torsion angles; correlations between esds in cell parameters are only used when they are defined by crystal symmetry. An approximate (isotropic) treatment of cell esds is used for estimating esds involving l.s. planes.

### Fractional atomic coordinates and isotropic or equivalent isotropic displacement parameters (Å^2^)

**Table:**

	*x*	*y*	*z*	*U*_iso_*/*U*_eq_	
Br1	0.62045 (2)	0.74137 (4)	0.56073 (3)	0.0982 (2)	
S1	0.33290 (3)	0.91269 (7)	0.18667 (5)	0.0519 (2)	
O1	0.34641 (11)	1.0325 (2)	0.13838 (14)	0.0677 (5)	
N1	0.28060 (11)	0.9785 (2)	0.23075 (16)	0.0525 (6)	
H1	0.267634	1.067206	0.217972	0.063*	
C1	0.53543 (14)	0.7926 (3)	0.4505 (2)	0.0576 (7)	
O2	0.30317 (11)	0.7841 (2)	0.13234 (14)	0.0629 (5)	
N2	0.22679 (11)	0.9777 (2)	0.33323 (17)	0.0579 (6)	
H2	0.223806	1.069690	0.323849	0.069*	
C7	0.25586 (12)	0.8997 (3)	0.2876 (2)	0.0486 (6)	
C2	0.52538 (15)	0.9312 (3)	0.4162 (2)	0.0664 (8)	
H2A	0.560352	1.000298	0.446974	0.080*	
O3	0.26161 (11)	0.76750 (17)	0.29256 (16)	0.0612 (5)	
C3	0.46344 (14)	0.9687 (3)	0.3360 (2)	0.0599 (7)	
H3	0.456463	1.062947	0.312127	0.072*	
C4	0.41168 (13)	0.8659 (3)	0.29115 (18)	0.0460 (6)	
C5	0.42201 (15)	0.7269 (3)	0.3267 (2)	0.0608 (8)	
H5	0.386886	0.657922	0.296706	0.073*	
C6	0.48436 (15)	0.6894 (3)	0.4068 (2)	0.0658 (8)	
H6	0.491670	0.595259	0.430921	0.079*	
C8	0.19943 (17)	0.9163 (4)	0.3988 (2)	0.0726 (9)	
H8A	0.215639	0.974924	0.458349	0.087*	
H8B	0.218898	0.820113	0.418427	0.087*	
C9	0.12066 (19)	0.9070 (4)	0.3534 (3)	0.0960 (12)	
H9A	0.103998	0.849364	0.293370	0.115*	
H9B	0.100819	1.003157	0.335194	0.115*	
C10	0.0955 (3)	0.8407 (5)	0.4230 (4)	0.1239 (17)	
H10A	0.044787	0.836946	0.391608	0.186*	
H10B	0.111388	0.898207	0.482141	0.186*	
H10C	0.114123	0.744633	0.439896	0.186*	

### Atomic displacement parameters (Å^2^)

**Table:**

	*U*^11^	*U*^22^	*U*^33^	*U*^12^	*U*^13^	*U*^23^
Br1	0.0584 (2)	0.1095 (4)	0.0856 (3)	0.01372 (19)	−0.00560 (18)	−0.0048 (2)
S1	0.0493 (4)	0.0394 (4)	0.0605 (4)	0.0027 (3)	0.0184 (3)	0.0051 (3)
O1	0.0721 (13)	0.0571 (12)	0.0766 (13)	0.0052 (10)	0.0354 (11)	0.0223 (11)
N1	0.0505 (12)	0.0283 (10)	0.0788 (16)	0.0045 (10)	0.0285 (12)	0.0093 (11)
C1	0.0395 (14)	0.070 (2)	0.0567 (16)	0.0047 (14)	0.0149 (13)	−0.0058 (15)
O2	0.0633 (11)	0.0485 (11)	0.0599 (11)	0.0019 (10)	0.0118 (10)	−0.0062 (9)
N2	0.0567 (14)	0.0405 (12)	0.0777 (16)	0.0001 (11)	0.0308 (13)	0.0032 (12)
C7	0.0330 (12)	0.0342 (14)	0.0648 (16)	−0.0019 (11)	0.0091 (12)	0.0005 (13)
C2	0.0507 (16)	0.064 (2)	0.075 (2)	−0.0164 (15)	0.0188 (15)	−0.0123 (17)
O3	0.0663 (13)	0.0297 (10)	0.0885 (14)	−0.0019 (9)	0.0349 (11)	0.0027 (9)
C3	0.0571 (17)	0.0459 (16)	0.0736 (19)	−0.0083 (14)	0.0262 (16)	0.0022 (15)
C4	0.0442 (13)	0.0360 (14)	0.0596 (16)	0.0011 (11)	0.0245 (12)	0.0003 (12)
C5	0.0492 (16)	0.0419 (16)	0.0746 (19)	−0.0030 (13)	0.0122 (14)	0.0007 (14)
C6	0.0560 (17)	0.0503 (16)	0.0736 (19)	0.0099 (15)	0.0125 (15)	0.0091 (16)
C8	0.077 (2)	0.066 (2)	0.077 (2)	0.0027 (18)	0.0361 (18)	0.0017 (18)
C9	0.089 (3)	0.084 (3)	0.133 (3)	0.004 (2)	0.066 (3)	0.025 (2)
C10	0.153 (4)	0.078 (3)	0.201 (5)	0.008 (3)	0.133 (4)	0.013 (3)

### Geometric parameters (Å, º)

**Table:**

Br1—C1	1.887 (3)	C3—C4	1.378 (4)
S1—O1	1.4207 (19)	C3—H3	0.9300
S1—O2	1.4225 (19)	C4—C5	1.372 (3)
S1—N1	1.634 (2)	C5—C6	1.379 (4)
S1—C4	1.763 (3)	C5—H5	0.9300
N1—C7	1.388 (3)	C6—H6	0.9300
N1—H1	0.8600	C8—C9	1.494 (4)
C1—C2	1.363 (4)	C8—H8A	0.9700
C1—C6	1.371 (4)	C8—H8B	0.9700
N2—C7	1.319 (3)	C9—C10	1.498 (5)
N2—C8	1.460 (4)	C9—H9A	0.9700
N2—H2	0.8600	C9—H9B	0.9700
C7—O3	1.228 (3)	C10—H10A	0.9600
C2—C3	1.374 (4)	C10—H10B	0.9600
C2—H2A	0.9300	C10—H10C	0.9600
			
O1—S1—O2	119.76 (13)	C3—C4—S1	119.8 (2)
O1—S1—N1	103.90 (12)	C4—C5—C6	120.1 (3)
O2—S1—N1	109.80 (12)	C4—C5—H5	119.9
O1—S1—C4	108.87 (12)	C6—C5—H5	119.9
O2—S1—C4	108.00 (12)	C1—C6—C5	119.2 (3)
N1—S1—C4	105.65 (11)	C1—C6—H6	120.4
C7—N1—S1	123.94 (17)	C5—C6—H6	120.4
C7—N1—H1	118.0	N2—C8—C9	113.9 (3)
S1—N1—H1	118.0	N2—C8—H8A	108.8
C2—C1—C6	121.1 (3)	C9—C8—H8A	108.8
C2—C1—Br1	119.8 (2)	N2—C8—H8B	108.8
C6—C1—Br1	119.1 (2)	C9—C8—H8B	108.8
C7—N2—C8	123.5 (2)	H8A—C8—H8B	107.7
C7—N2—H2	118.3	C8—C9—C10	111.7 (3)
C8—N2—H2	118.3	C8—C9—H9A	109.3
O3—C7—N2	124.7 (3)	C10—C9—H9A	109.3
O3—C7—N1	120.4 (3)	C8—C9—H9B	109.3
N2—C7—N1	114.8 (2)	C10—C9—H9B	109.3
C1—C2—C3	119.8 (3)	H9A—C9—H9B	107.9
C1—C2—H2A	120.1	C9—C10—H10A	109.5
C3—C2—H2A	120.1	C9—C10—H10B	109.5
C2—C3—C4	119.7 (3)	H10A—C10—H10B	109.5
C2—C3—H3	120.1	C9—C10—H10C	109.5
C4—C3—H3	120.1	H10A—C10—H10C	109.5
C5—C4—C3	120.0 (2)	H10B—C10—H10C	109.5
C5—C4—S1	120.1 (2)		

### Hydrogen-bond geometry (Å, º)

**Table:**

*D*—H···*A*	*D*—H	H···*A*	*D*···*A*	*D*—H···*A*
N1—H1···O3^i^	0.86	1.94	2.791 (3)	172
N2—H2···O2^i^	0.86	2.24	2.998 (3)	147
N2—H2···O3^i^	0.86	2.64	3.351 (3)	141

Symmetry code: (i) −*x*+1/2, *y*+1/2, −*z*+1/2.
